# Urolithin Metabotypes can Anticipate the Different Restoration of the Gut Microbiota and Anthropometric Profiles during the First Year Postpartum

**DOI:** 10.3390/nu11092079

**Published:** 2019-09-03

**Authors:** Adrián Cortés-Martín, María Romo-Vaquero, Izaskun García-Mantrana, Ana Rodríguez-Varela, María Carmen Collado, Juan Carlos Espín, María Victoria Selma

**Affiliations:** 1 Laboratory of Food and Health, Research Group on Quality, Safety and Bioactivity of Plant Foods, CEBAS-CSIC, 30100 Murcia, Spain; 2 Group of Lactic Bacteria and Probiotics, Department of Biotechnology, IATA-CSIC, 46980 Valencia, Spain; 3 Primary Health Care Center of Bétera, 46980 Valencia, Spain

**Keywords:** ellagitannins, polyphenols, gut microbiome, postpartum, lactation, body mass index, gut dysbiosis

## Abstract

The metabolism of dietary polyphenols ellagitannins by the gut-microbiota allows the human stratification in urolithin metabotypes depending on the final urolithins produced. Metabotype-A only produces urolithin-A, metabotype-B yields urolithin-B and isourolithin-A in addition to urolithin-A, and metabotype 0 does not produce urolithins. Metabotype-A has been suggested to be ‘protective’, and metabotype-B dysbiotic-prone to cardiometabolic impairments. We analyzed the gut-microbiome of 40 healthy women and determined their metabotypes and enterotypes, and their associations with anthropometric and gut-microbial changes after 3 weeks, 4, 6, and 12 months postpartum. Metabotype-A was predominant in mothers who lost weight (≥2 kg) (75%) versus metabotype-B (54%). After delivery, the microbiota of metabotype-A mothers changed, unlike metabotype-B, which barely changed over 1 year. The metabotype-A discriminating bacteria correlated to the decrease of the women’s waist while some metabotype-B bacteria were inversely associated with a reduction of body mass index (BMI), waist, and waist-to-hip ratio. Metabotype-B was associated with a more robust and less modulating microbial and anthropometric profiles versus metabotype-A, in which these profiles were normalized through the 1-year follow-up postpartum. Consequently, urolithin metabotypes assessment could be a tool to anticipate the predisposition of women to normalize their anthropometric values and gut-microbiota, significantly altered during pregnancy and after childbirth.

## 1. Introduction

During a healthy pregnancy, body fat increases followed by a reduction of insulin sensitivity and the elevation of circulating cytokines that are thought to drive obesity-associated metabolic inflammation [1]. The gut microbiota of pregnant women is profoundly altered with a vast expansion of alpha-diversity and a general increase of different bacterial groups, which persists at least 1 month after delivery [2,3]. This change induces higher adiposity and insulin insensitivity, which resembles obesity-associated metabolic syndrome traits. Some authors suggested the benefit of these changes in gestation to support the energetic demands and future lactation [3]. However, pregnancy per se is a major contributor to the obesity epidemic among women, through the retention of weight gained during pregnancy [4,5]. Estimates of postpartum weight retention (PPWR) vary, but up to 20% of women can retain more than 5 kg after one year postpartum. Additionally, PPWR has been associated with an increased risk of many adverse outcomes in a subsequent pregnancy, independently of the woman’s initial body mass index (BMI) [4]. Therefore, a clear understanding of the PPWR risk factors is needed to face the problem, and the microbiota seems to play an important role [4,5].

In a healthy population, the gut microbiota composition varies dramatically among individuals [6]. Three enterotypes, driven by *Bacteroides*, *Prevotella*, and *Ruminococcus,* were proposed to cluster the population according to their microbiome and were suggested to be functional markers by Arumugan et al. [7]. Other authors proposed only two enterotypes, *Prevotella* and *Bacteroides*. These two clusters had been associated with long term diets, particularly *Bacteroides* enterotype, which was related to protein and animal fat versus *Prevotella,* more often associated with the intake of carbohydrates [8]. However, BMI, gender, or age cannot explain the observed enterotypes [7,9]. Recently, the enterotype concept has been under debate, and recent studies proposed the existence of microbial community gradients rather than distinct enterotypes [9,10].

Recently, the metabolism of some polyphenols by specific gut microbial groups has given rise to the so-called ‘polyphenol gut metabotypes’, which could indirectly reflect inter-individual differences in the status of the individuals’ gut microbiome [11,12]. This is the case of the gut microbiota-mediated metabolism of the polyphenols ellagitannins and ellagic acid to yield urolithins. Three urolithin metabotypes have been described so far depending on the final urolithins produced, i.e., metabotype A (urolithin metabotypes (UM)-A) produces only urolithin A (Uro-A), metabotype B (UM-B) yields urolithin B (Uro-B) and IsoUro-A in addition to Uro-A, and metabotype 0 (UM-0) does not produce these final urolithins [13,14]. At present, only two bacterial genera from the human gut, belonging to the Coriobacteriaceae family, have been identified as urolithin producers, i.e., *Gordonibacter,* an urolithin C producer that positively correlates with Uro-A and UM-A, and *Ellagibacter isourolithinifaciens*, an IsoUro-A producer that positively correlates with IsoUro-A and UM-B [15,16,17,18]. A recent study has identified that UMs and enterotypes are not a coincident [19]. Remarkably, a higher gut microbiome alpha-diversity (Chao 1 index), proinflammatory microorganisms including methanogenic Archaea from the Euryarchaeota phylum (*Methanobrevibacter* and *Methanosphaera* genera), as well as the Gammaproteobacteria class were increased in UM-B versus UM-A, which could suggest that UM-B is a gut dysbiosis-prone metabotype [19]. Previous studies have shown that overweight-obese individuals belonging to UM-B exhibited increased cardiovascular disease (CVD) risk (higher blood total cholesterol, low-density lipoprotein (LDL)-cholesterol and oxidized LDL levels among others), whereas UM-A seemed to be a protective metabotype against CVD risk factors [20,21]. Preliminary observations also associated UM-B with obesity and gastrointestinal pathologies driven by gut dysbiosis [13,22]. However, this has not been unequivocally confirmed yet [23].

The distribution of UMs in the healthy population and their association with aging has previously been examined in a large cohort (*n* = 839) aged from 5 to 90 years [23]. However, the distribution of UMs is still unknown in postpartum. Besides, most studies have focused on the gut microbiota during pregnancy, especially in association with cardiometabolic risk factors [1,2,3,4,5]. However, the long-term evolution of the postpartum microbiota is poorly understood. The possible contribution of UMs in this microbiota reshaping process is also unknown. In the present study, we aimed to (i) characterize the UMs of 40 healthy women 3 weeks after delivering a healthy baby (where the microbiota is still similar to pregnancy), (ii) evaluate the stability of these UMs over one year, and (iii) estimate the possible association of maternal enterotypes and UMs with the restoration of both anthropometric values (BMI, waist and hip circumferences and waist-to-hip ratio) and gut microbiome ecology.

## 2. Materials and Methods

### 2.1. Study Design and Diet

Healthy women (*n* = 40), one week after vaginal (*n* = 28) or cesarean (*n* = 12) delivering a healthy baby and with breastfeeding during the neonatal period, were recruited in a public health center (Betera, Spain). One multipara woman was also included. Exclusion criteria included dietary supplementation (prebiotic and probiotic supplements) and drug administration (antibiotics) during one-year after giving birth. The pilot was included in the Spanish National Project AGL2015-64124-R (‘*PolyMicroBio*’) and was conducted in line with the Helsinki Declaration and ethical principles for medical research involving human subjects (Seoul, Korea, 2008). The protocol was revised and approved by the ethics committees from both the Servicio Valenciano de Salud (reference 52327) (Valencia, Spain) and the Spanish National Research Council (CSIC, Spain) (reference AGL2015-64124-R). Written informed consent was obtained from all the participants.

**Figure 1:**
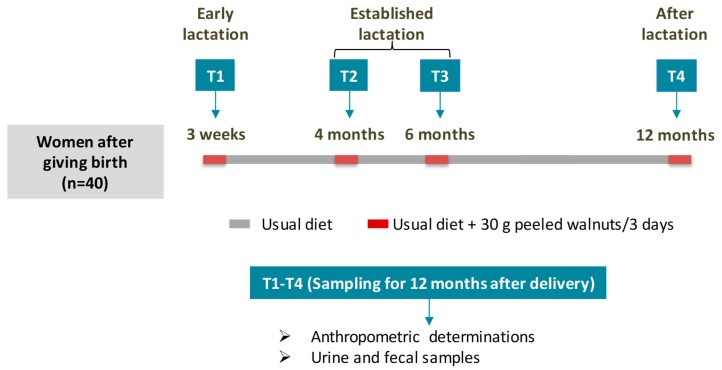
Study design of mothers through postpartum.

Three different stages after childbirth were analyzed (Figure 1) that coincided with the pediatric/clinical reviews established in the official health calendar: “Early lactation” (T1: 3 weeks after delivery and breastfeeding), “Established lactation” (T2 and T3: 4 and 6 months after delivery respectively, and breastfeeding) and “After lactation” period (T4: 12 months after delivery and formula-feeding). The volunteers continued with their usual physical activity and diet throughout the study and their diet was not restricted. The physical activity intensity of the volunteers was light and consisted of walking (3.4 ± 3.2 h/week). The eating pattern of the volunteers was characteristically Mediterranean according to the PREDIMED (PREvención con DIeta MEDiterránea) validated test [24]. The adherence rate to a healthy Mediterranean diet was high (≥9) for all volunteers. For UMs stratification, and using a protocol previously described [23], mothers consumed an ellagitannin-rich food (30 g of peeled walnuts) daily for three days in the four specific-time-points after childbirth (T1–T4, Figure 1). The peeled walnuts used in the study were kindly supplied by Borges S.A. (Reus, Tarragona, Spain).

### 2.2. Anthropometric Determinations

Maternal anthropometric data, including waist, hip, and weight as well as BMI (kg/m^2^) was obtained at each time point (T1 to T4, Figure 1). Height without shoes was measured in the first visit only (3 weeks after delivery). Waist and hip circumferences were measured in an erect position midway between the iliac crest and the lower costal margin and at the level of the pubic symphysis respectively, using a non-expandable tape. Normal weight was defined as BMI < 25 kg/m^2^, overweight as BMI ≥ 25 kg/m^2^ and < 30 kg/m^2^, and obesity as BMI ≥ 30 kg/m^2^ [25]. Weight loss during the first year after delivery was also recorded in each visit.

### 2.3. Sampling Procedure and Determinations

Maternal urine and fecal samples were collected after consuming walnuts for three days in the four selected time-points after delivery (T1-T4, Figure 1). Samples were aliquoted and frozen at −80 °C for further analysis. Urolithins were determined in urine samples (T1–T4, Figure 1) by Ultra Performance Liquid Chromatography–Electro Spray Ionization–Quadrupole Time of Flight–Mass Spectrometry (UPLC-ESI-qToF-MS), as previously described [26]. The individuals were stratified according to their different capacity to metabolize ellagitannins and ellagic acid derivatives into urolithins, i.e., UMs (UM-A, UM-B, and UM-0), as previously described [13].

Maternal gut microbiota analysis was performed by 16S ribosomal RNA (16S rDNA) sequencing. Metagenomics sequencing library preparation Illumina (Illumina Inc., San Diego, CA, USA), as well as sequence processing and taxonomic classification of gut microbiota, were achieved as described previously [19]. Paired-end sequencing with a read length of 2 × 300 bp was obtained using a Illumina^®^ MiSeq^®^ Reagent kit v3 (MS-102-3001) on a MiSeq-Illumina^®^ platform (FISABIO sequencing service, Valencia, Spain). The clustered sequences were utilized to construct Operational Taxonomic Units (OTUs) tables with 97% identity, and representative sequences were classified into the respective taxonomical level from phylum to genus using the Ribosomal Database Project (RDP) classifier. Analyses with RDP pipeline (http://pyro.cme.msu.edu/) involved 16S rRNA gene sequence alignment (Aligner), 16S rRNA gene sequence clustering (Complete Linkage Clustering) and alpha-diversity indexes (Shannon Index and Chao1 estimator). Alpha-diversity (Chao1 and Shannon indexes), based on a randomly selected 21,181 reads per sample, were used to estimate the samples’ richness and diversity. Enterotypes were identified by differences in relative abundances of genera *Bacteroides, Prevotella*, and *Ruminococcus,* as previously described [7,9].

### 2.4. Statistical Analysis

Statistical analysis was performed using the SPSS Software, version 23.0 (SPSS Inc., Chicago, IL, USA). Mothers were grouped into UMs and enterotypes using principal component analysis (PCA) and hierarchical clustering analysis (HCA) via R commander using ‘ggbiplot’ graphics [19]. We applied a multinomial logit model to evaluate changes in UMs and enterotype distribution in mothers over the first year after childbirth (T1–T4, Figure 1). Additionally, microbial and anthropometric changes were associated by Spearman’s correlation. Parameters with skewed distribution were transformed into their logarithm for analysis. Maternal changes of anthropometric values, microbial diversity and richness, and microbiota relative abundance were evaluated by a covariance model (ANCOVA) for repeated measures over the first year after childbirth. The Linear Discriminant Analysis (LDA) Effect Size (LEfSe) algorithm with the online interface Galaxy (http://huttenhower.sph.harvard.edu/galaxy/root) were both used to identify taxa with differentiating abundance among UMs as well as maternal microbiota changes in the first year after childbirth. LEfSe identified features that were statistically different among the different groups and performed non-parametric factorial Kruskal–Wallis sum-rank tests and Linear Discriminant Analysis (LDA) to determine whether these features were consistent concerning the expected behavior of the different UMs. Statistical significance was set at * *p* < 0.05, ** *p* < 0.01, and *** *p* < 0.001. A trend towards significance was also acknowledged when 0.1 > # *p* > 0.05.

Potential microbial functions were identified by PICRUst v0.9.0 (http://picrust. github.io/picrust/) as described elsewhere [27]. Following PICRUst analysis, the potential microbial functions associated with UMs and postpartum times were identified by LEfSe. A LDA score was generated using linear discriminate analysis for KEGG (Kyoto Encyclopedia of Genes and Genomes) pathways.

## 3. Results

### 3.1. Distribution of Microbiome Enterotypes and Urolithin Metabotypes (UMs) of the Mothers Over One Year After Delivery

The mothers who completed the four stages of the study (*n* = 32) were clustered into enterotypes and UMs (Table 1). Most of the mothers were grouped into the enterotype 3 (*Ruminococcus*-type, 88%) at early lactation (T1) and remained invariable throughout the study (Table 1). The *Prevotella* to *Bacteroides* ratio did not differ throughout the study either. Regarding UMs, UM-A and UM-B were equally abundant (50% mean value) whereas UM-0 was absent at T1 (Table 1). UMs distribution for UM-0, UM-B, and UM-A was maintained through T1 to T2, and slightly changed at T3 (0%, 53%, 47%, respectively) but especially at T4, after lactation (0%, 60%, 40%, respectively) (Table 1). The increase in the percentage of normoweight mothers with UM-A overtime was concomitant with a decrease in the percentage of overweight mothers with UM-B. The percentage of obese mothers remained stable throughout the study (Table 1).

### 3.2. Changes of Anthropometric Values over One Year after Giving Birth

Table 2 shows significant and gradual reductions in BMI, waist, hip, and waist-to-hip ratio (WHR) in the mothers from T1 to T4 after giving birth. However, while BMI reached mean normoweight values, the mean waist and WHR values were above 80 and 85 respectively, even 12 months after delivery (Table 1). Only 66% of mothers lost weight (≥2 kg), while 24% did not lose weight, and 10% even gained weight (≥2 kg) during the 12 months after childbirth (data not shown). Clustering of the mothers according to their UMs, revealed that the percentage of mothers who lost weight (≥2 kg) was higher in the UM-A group (75%) versus UM-B (54%) (data not shown), and only the UM-A group showed changes in BMI and waist steadily throughout the three stages: early lactation (T1), established lactation (T2 and T3) and after lactation (T4). Accordingly, mean normoweight BMI values were observed only in the UM-A group. Furthermore, WHR reduction was only significant in UM-A women (Table 2). Clustering of the mothers who completed the four stages of the study (*n* = 32) according to natural (*n* = 24) or cesarean (*n* = 8) delivery, showed significant and gradual reductions in BMI and waist in both groups (*p* < 0.05) from T1 to T4 after giving birth. However, hip and WHR reductions were only significant in the cesarean delivery group (*p* < 0.05) (data not shown).

### 3.3. Alpha-Diversity and Composition of the Maternal Gut Microbiota for One Year after Delivery

Shannon index of the mothers’ gut microbiota was not significantly reduced during postpartum regardless of UMs (Table 2). The Shannon index in the UM-B group tended to be higher versus UM-A after twelve months of delivery (*p* = 0.083). At T1, the Chao1 index of the mothers’ gut microbiota (2472 ± 132) was significantly reduced in all the mothers, without clustering into metabotypes (*p* = 0.004), or in those with UM-A (*p* = 0.011). Although the UM-B group followed the same trend, index reduction was only marginally significant (*p* = 0.078) (Table 2). After lactation (T4), the differences in the Chao1 index between UM-A and UM-B became closer to the significance (*p* = 0.161) (data not shown).

**Figure 2:**
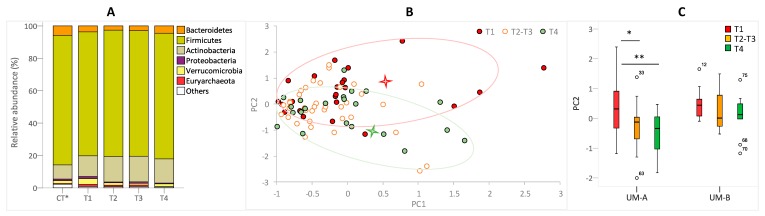
Microbial taxonomic composition in fecal samples of mothers through postpartum. (**A**) The bars show the mean proportion at the phylum level. T1: early lactation stage (3 weeks after delivery); T2 and T3: established lactation stage (4 and 6 months after delivery, respectively); T4: after lactation stage (12 months after delivery); Control (CT): healthy adult cohort (*n* = 249) previously published [18]. (**B**) Principal component analysis (PCA) and clustering analysis that shows differences in fecal microbiome through postpartum. (**C**) Box plots of second axes of the PCA (PC2) through postpartum in UM-A and UM-B groups.

**Figure 3:**
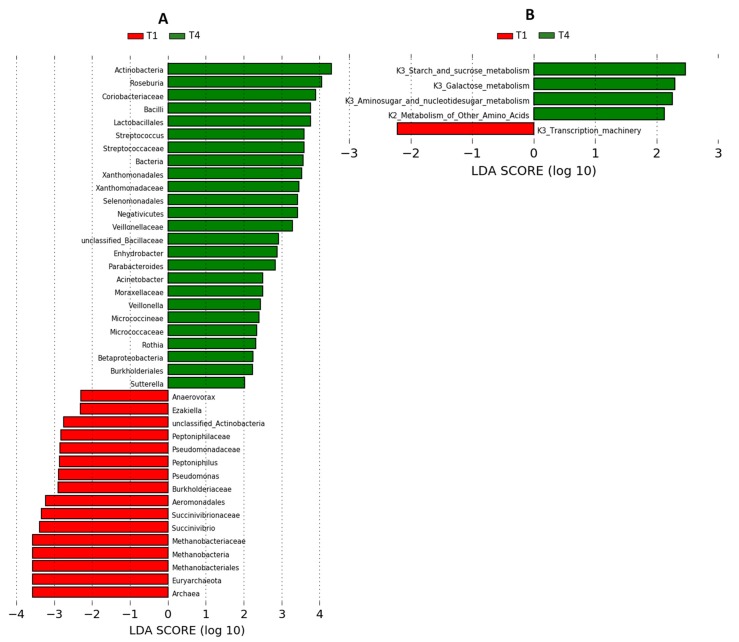
Linear discriminant analysis (LDA) effect size (LEfSe) analysis of gut microbiota that shows different abundances through postpartum in (**A**) maternal fecal microbiome and (**B**) inferred functional capacity of the fecal microbiome. Positive LDA scores (green bars) are enriched after the lactation stage (T4, 12 months after delivery) while negative LDA scores (red bars) are enriched in the early lactation stage (T1, 3 weeks after delivery). The microbial taxa and functions shown in the Figure have a LDA score higher than 2.

Maternal gut microbiota was significantly modified throughout the postpartum. Between T2 and T3, when the lactation is thoroughly established, the gut microbiota was very similar to each other but different from the microbiota of T1 (early lactation) (Figure 2A). The Actinobacteria phylum (*p* = 0.034) and the Bacilli class, including *Lactobacillus* and *Streptococcus*, were increased in T2 and T3 versus T1. In contrast, the higher abundance of Verrucomicrobia (*p* = 0.034) together with its genus *Akkermansia* (*p* = 0.016) observed at T1 disappeared after lactation (T4) (Figure 2A). At T4, the Actinobacteria abundance remained higher than in T1 but similar to T2 and T3 (Figure 2A). In contrast, the abundances of the Euryarchaeota phylum (*p* = 0.004) and its genus *Methanobrevibacter* (*p* = 0.001) were reduced at T4, whereas total bacteria (*p* = 0.035) were increased, becoming significantly different from those of the early lactation stage (T1) (Figure 2A). The LEfSe analysis confirmed the differences between T1 and T4, especially in the methanogenic Archaea (Methanobacteria) and the methanotrophs (*Pseudomonas* and Aeromonadales from Gammaproteobacteria class) versus total bacteria, Actinobacteria (Coriobacteriaceaea family) and Bacilli (*Streptococcus* and *Lactobacillus*), which were predominant at T4 (Figure 3A). Accordingly, the PCA plot of the gut microbiome illustrated differences of the early lactation stage (T1) from both the established lactation stage (T2 and T3) (*p* = 0.016) and after the lactation stage (T4) (*p* = 0.008), but only in the second principal component (PC2), where the main drivers were Archaea versus Actinobacteria (Figure 2B). Figure 2C shows the differences in PC2 among mother UMs throughout the postpartum.

**Figure 4:**
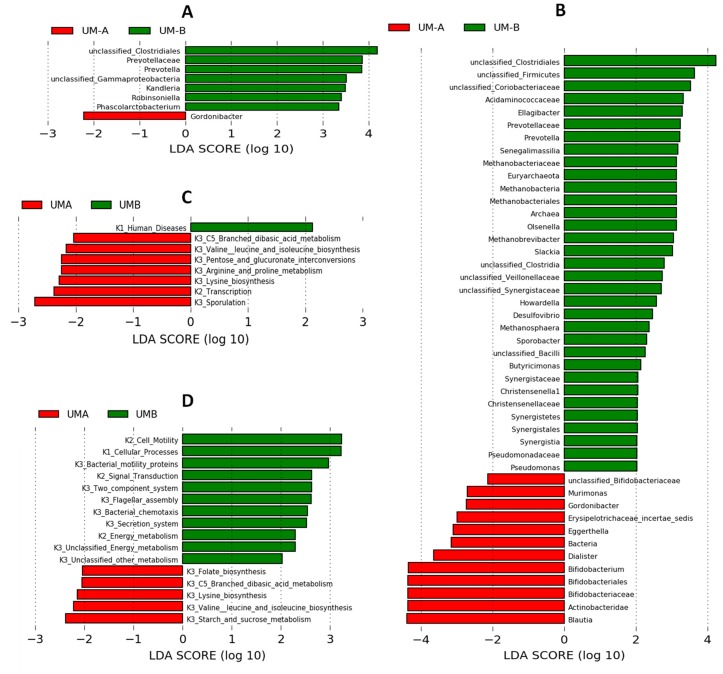
Linear discriminant analysis (LDA) effect size (LEfSe) analysis of gut microbiota that shows different abundances between urolithin metabotypes (Ums) in the maternal fecal microbiome (**A**,**B**), and the inferred functional capacity of the fecal microbiome (**C**,**D**) at T1 (**A**,**C**) and T4 (**B**,**D**). Positive LDA scores (green bars) are enriched in UM-B while negative LDA scores (red bars) are enriched in UM-A. The microbial taxa and functions shown in the figure have a LDA score higher than 2.

When the mothers at T1 were clustered into UMs, the differences between UM-A and UM-B in the microbial composition affected a minimal number of bacteria including *Gordonibacter* and *Prevotella,* which predominated in UM-A and UM-B, respectively (Figure 4A). The relative abundance (%) changed in a higher number of bacterial groups in UM-A compared to UM-B during postpartum. Consequently, differences between UM-A and UM-B increased from T1 to T4 (Figure 4A, B). The LEfSe analysis at T4 confirmed the differences between UM-A and UM-B at genus, family, class, and order levels (Figure 4B). A higher abundance of *Prevotella*, the methanogenic Archaea including *Methanobrevibacter* and *Methanosphaera* genera, and the methanotrophs (*Pseudomonas* from Gammaproteobacteria class) and some Coriobacteriales (*Olsenella, Senegalimassilia, Slackia*, and unclassified *Coriobacteriacea*) were observed in UM-B versus UM-A (Figure 4B). In contrast, a higher abundance of total bacteria, other Coriobacteriales (*Gordonibacter* and *Eggerthella*) as well as *Bifidobacterium* and *Blautia* was found in UM-A.

### 3.4. The Functionality of the Maternal Gut Microbiota During the First Year after Delivery

Since the microbial composition of the mothers changed over one year after giving birth, we next investigated how the functionality of the microbiota was affected by using amplicon sequencing predictions. Amplicon sequencing functional content was predicted from marker genes (16S rRNA), and LDA was performed. After childbirth, we observed progressive changes in the maternal microbiota functions from T1 to T3, characterized by a decrease in transcription processes and an increase in the carbohydrate metabolism (galactose, starch and sucrose metabolisms, amino sugar and nucleotide sugar metabolisms) and in the metabolism of other aminoacids (data not shown). At T4, these microbial functions remained higher than in T1 but similar to T2 and T3 (Figure 3B).

The clustering of the mothers into UMs revealed minimal differences in the functionality of the gut microbiota between UM-A and UM-B at T1. At that time, UM-A was predicted to enrich functions of the microbiome that included the biosynthesis and metabolism of some aminoacids as well as carbohydrate metabolism (Figure 4C). During postpartum, in addition to a decrease in transcription processes and an increase in both carbohydrate and aminoacid metabolisms, we also observed in UM-A an increase in phenylpropanoid biosynthesis and cyaminoacid metabolism, the latter within the metabolism of other aminoacids (data not shown). Besides, carbon fixation pathways and arginine and proline metabolisms specifically decreased in UM-A. In contrast, we did not observe specific changes in functionality during postpartum associated with UM-B (data not shown). Consequently, the differences between UM-A and UM-B increased throughout the postpartum period, being more evident after 12 months (T4) (Figure 4D). At that time, UM-A was predicted to enrich functions of the microbiome that included vitamin and aminoacid biosynthesis (including folate, lysine, valine, leucine and isoleucine biosynthesis) and carbohydrate metabolism (starch and sucrose metabolism and C5-branched dibasic acid metabolism). In contrast, UM-B was predicted to result in a gut microbiome with functions characterized by the increased flagellar assembly, cell motility, bacterial chemotaxis, and two-component systems related to cell motility and virulence. It is worth noting that energy metabolism and unclassified energy metabolism for energy harvest were also increased in UM-B (Figure 4D).

**Figure 5:**
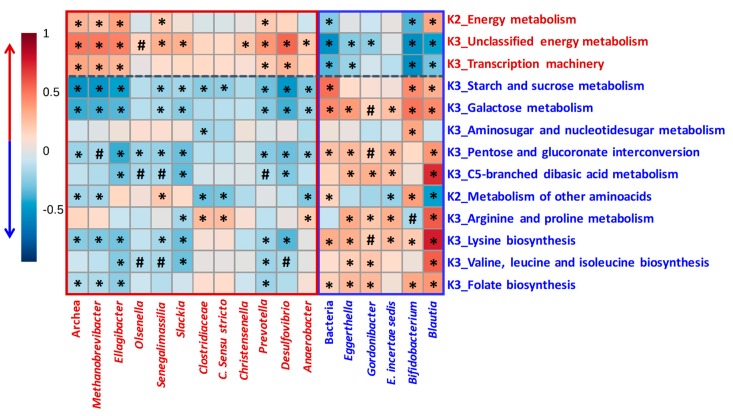
Spearman’s correlations heatmap between specific microbial groups and microbiota functions through postpartum. *: Spearman’s correlation values at *p* < 0.05 and **#**: 0.05 > *p* > 0.1. Microbial groups and functions augmented in UM-B (red color) and UM-A (blue color) were included. The strongest positive (r = 1) and negative correlations (r = −1) are indicated in red and blue, respectively.

Figure 4 shows significant correlations between some microbial functions and microbial groups of mothers through postpartum. Energy metabolism, which was increased in UM-B, was positively associated with an increased abundance of several microbial groups in UM-B such as Archaea, *Methanobrevibacter*, and bacteria as *Ellagibacter, Senegalimassilia*, and *Prevotella*. In contrast, energy metabolism was negatively associated with bacteria kingdom and *Bifidobacterium* genus, which predominated in UM-A versus UM-B at T4 (Figure 5). Unclassified energy metabolism and transcription machinery, which were also augmented in UM-B, were positively associated with UM-B discriminating genera and negatively associated with UM-A discriminating genera (Figure 5). Interestingly, several bacterial groups augmented in UM-A (total bacteria, *Eggerthella, Gordonibacter*, *Erysipelotrichaceae incertae sedis, Bifidobacterium* and *Blautia*) positively correlated with many of the microbial functions enriched in UM-A (Figure 5).

### 3.5. Associations of Gut Microbial and Anthropometric Changes of the Mothers Over One Year after Delivery

**Figure 6:**
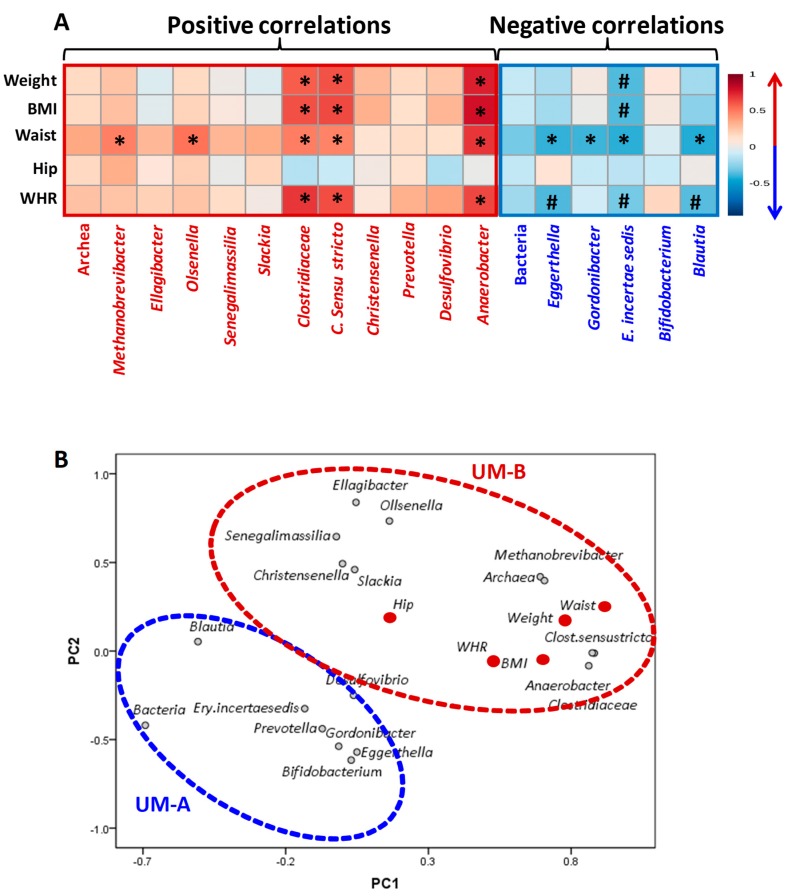
Spearman’s correlations heatmap (**A**) and principal component analysis (**B**) of changes through postpartum in microbial and anthropometric values. Changes in microorganisms and anthropometric values through postpartum were obtained by subtracting the initial values (T1, early lactation stage) from the finals (T4, after lactation stage) *: Spearman’s correlation values at *p* < 0.05 and #: 0.05 > *p* > 0.1. The strongest positive (r = 1) and negative correlations (r = −1) are indicated in red and blue, respectively. The microbial groups increased in UM-B (red color) and UM-A (blue color) were included in the PCA plot.

Spearman’s correlation showed associations between microbial and anthropometric changes from T1 to T4 (Figure 6A), and PCA was plotted (Figure 6B). The reduction of the Clostridiaceae family and its genera *Clostridium sensu stricto* and *Anaerobacter,* which only occurred in the UM-A group, was associated with a reduction of several anthropometric values (waist, BMI, WHR, and weight) (Figure 6A, B). The decrease of *Methanobrevibacter* and *Olsenella*, which were reduced in UM-A *versus* UM-B, was associated with the decrease in mothers’ waist during postpartum (Figure 6A). In contrast, the increase of *Eggerthella, Gordonibacter*, *Erysipelotrichaceae incertae sedis*, and *Blautia,* which were significantly augmented in UM-A versus UM-B, was associated with the decrease of the mother’s waist (Figure 6A).

## 4. Discussion

Postpartum, along with pregnancy period, constitute a crucial period in women’s lives in which the risk of obesity is a reality. Several studies have tried to understand PPWR risk factors where pregnancy weight gain, lifestyle, and gut microbiota, among others, seem to play an important role [4,5]. A recent meta-analysis has shown that light to moderate physical activity itself may not be sufficient to induce weight loss after birth [28]. Contrary, the combination of a healthy diet and physical activity interventions reduced PPWR in women of any BMI [28]. In the present study, a healthy Mediterranean diet and light physical exercise were common in all volunteers. Therefore, the impact of diet and exercise and their link to maternal weight postpartum could not be investigated. There is no clear evidence of the link between cesarean delivery and maternal weight retention [29,30]. In the present study, less difficulty in restoring the normal anthropometric profile during postpartum was found in cesarean versus vaginal delivery mothers. Nevertheless, the number of cesarean delivery participants (*n* = 8) is perhaps not high enough for meaningful results and needs further confirmation in larger cohorts.

Gut microbiota and UMs distribution in mothers were changing during postpartum to resemble the distribution in the general population previously described [19]. The decrease in the percentage of overweight mothers with UM-B was concomitant with the increase of normoweight mothers with UM-A over time. Although the correlation between UM-B and obesity cannot be unequivocally established [23], however, the results of the present study suggest that the UM-B dysbiosis-prone metabotype could be a potential contributor to obesity. We also confirm here that pregnancy is a risk factor for women to become overweight and obese, as many women did not lose weight during postpartum as previously reported [5]. However, our study showed that the percentage of mothers who lost weight was higher in the UM-A group versus UM-B. These results suggest that UM-A mothers could have less difficulty in restoring the normal anthropometric profile during postpartum and thus, the gut microbiota associated with UMs could have, directly or indirectly, a significant role in these differences.

Progressively more studies are investigating the biological importance of enterotypes, but the evidence is still limited, and the existence of a smooth gradient rather than a discrete distribution in the gut microbiota (three or two enterotypes) has been proposed [7,8,9,10,31]. In the present study, enterotype distribution did not change throughout the 12-months postpartum period. Our results agree with those reported in mothers during pregnancy, where enterotyping did not predict differences within trimesters [3]. Therefore, UMs could be a better estimator than enterotypes of the gut microbiome reshaping during postpartum.

The Shannon index at early lactation (T1) was similar to that of the non-pregnant healthy cohort previously published (4.3 ± 0.4) [19], and also similar to that of a mother’s group during the perinatal period (4.4 ± 0.5) (1 month before and after delivery) [31]. The Shannon index was not significantly reduced during postpartum regardless of UMs. Therefore, the Shannon index seems not to reflect the changes of the gut microbiota during pregnancy and postpartum. In contrast, the Chao1 index at T1 (2472.2 ± 132.2) was higher than that of the non-pregnant healthy cohort (1971.5 ± 633.4). Furthermore, the Chao1 index was significantly reduced during postpartum when we considered all the mothers together or only those of the UM-A group, resembling the richness of a healthy cohort [19]. Crussell et al. [32] also showed that OTU richness decreased from late pregnancy (third trimester) to eight months postpartum. Furthermore, a higher bacterial richness and strong correlations between pro-inflammatory taxa and metabolic/inflammatory variables were detected in gestational *diabetes mellitus* (GDM) patients through pregnancy [33]. Therefore, the reduction of richness seems to be a natural process throughout the postpartum, even healthy, but it does not occur with the same facility in all mothers. Chávez-Carbajal et al. [34] reported higher Chao1 index in non-pregnant obese women and obese-metabolic syndrome women than in a healthy control group [34]. Therefore, richness (Chao1 index) rather than diversity (Shannon index) may better reveal the changes in the gut microbiota during pregnancy and postpartum and indirectly, the changes in BMI and metabolic status. A previous study showed that UM-0 individuals are characterized by having less richness than UM-A, and this, in turn, less than UM-B [19]. Therefore, this may justify the absence of UM-0 and the increased abundance of UM-B versus UM-A in mothers at the early lactation stage (T1) where richness is higher than in mothers 12 months after childbirth (T4).

At early lactation (T1), where the microbiota is similar to late pregnancy [2,3], we observed an increase of several bacterial groups concerning a healthy cohort [19]. An increased abundance of Actinobacteria, Proteobacteria, and Verrucomicrobia phyla as well as their families Bifidobacteriaceae and Coriobacteriaceae and the genus *Akkermansia* was observed in mothers, according to previous results in women at late pregnancy or early postpartum [2,3]. An increase in the abundance of Proteobacteria in the mother’s gut has previously been associated with the adaptations of the maternal digestive tract to late pregnancy, which is characterized by weakened gut barriers against bacterial growth and increased permeability [35]. The Actinobacteria phylum gradually increased during pregnancy and early lactation and was mainly attributed to an increase in the abundance of bacteria from the family Coriobacteriaceae and Bifidobacteriaceae. This is in line with a previous study in sows during pregnancy and lactation where authors suggested that Coriobacteriaceae were likely associated with the progression of pregnancy [35]. In a non-pregnant cohort, Coriobacteriaceae, whose abundance was the highest in UM-B, was positively correlated with blood total-cholesterol, LDL-cholesterol, and BMI [19]. Accordingly, Koren et al. [3] suggested that the microbial composition at late pregnancy and early postpartum, although similar to that reported in non-pregnant adults with metabolic syndrome, was beneficial to support the growth of the fetus and to prepare the mother for energetic demands of lactation [3]. Between four and six months after delivery, where lactation is thoroughly established, the microbiota was very similar to each other, as described before [36], but different to those of early lactation and a control cohort [19]. Consequently, the gut microbiome composition was progressively changing through the postpartum period. The intestinal microbiota of healthy mothers after six months of giving birth has been scarcely studied. LEfSe analysis confirmed the differences between T1 and T4 stages, especially in methanogenic Archaea versus bacteria and Bacilli class (*Streptococcus* and *Lactobacillus*) predominant after the lactation stage. Similar results were shown in a recent study where the maternal gut microbiota of sows was enriched in *Streptococcus* throughout the stages of gestation, lactation, and the empty phase [35]. Interestingly, *Streptococcus thermophilus* has been associated with maintenance of mucosal barrier function and reduction of bacterial translocation and thereby reducing immune stimulation and associated inflammation [34]. When the mothers were clustered into UMs, the UM-A group modified their microbiota during postpartum as mentioned before, differing more and more from those of UM-B, while those of the UM-B barely modified their microbiota. Consequently, after 12 months, the microbial profile of the mothers differed mainly depending on which metabotype they belonged, similar to the microbial differences found between UM-A and UM-B in healthy, non-pregnant, subjects [19].

The significant role of the gut microbiota plays in energy intake and harvesting from the diet has been previously suggested in pregnancy [32,35]. It is currently accepted that, like in obesity, there is a pregnancy gut microbiota harvesting higher energy from soluble dietary fiber through fermentation, producing more short-chain fatty acids (SCFA), and influencing in this manner the host energy metabolism, which has been hypothesized as beneficial in the context of pregnancy [32,35]. After delivery, from T1 to T2-T3, we found progressive changes in motherly microbiota functions dealing with energy harvest. At T4, these microbial functions remained higher than in T1 but similar to T2 and T3. This suggests that the gut microbiota of mothers during the first year of life of the child and not only during pregnancy has a higher capacity for energy harvest. When the mothers were grouped into UMs, the different predicted functions of the gut microbiome between UM-A and UM-B suggest that UM-B results in microbiota with increased capacity for energy metabolism while UM-A results in microbiota with increased capacity for SCFA, vitamin and aminoacid biosynthesis. Significant correlations between several bacterial groups and functional activities increased in both UMs, confirmed these associations. Among others, Archaea and *Methanobrevibacter,* predominant in UM-B, positively correlated with energy metabolism probably because they are methanogens. In a previous study, more abundant metabolic pathways related to amino acids, amino sugars, as well as glycolysis/gluconeogenesis were found in gut bacteria of healthy lean women than in obese and obese-metabolic syndrome women [34], which was in agreement with the results observed for our UM-A group. Interestingly, some of UM-A discriminating bacteria were associated with the decrease of mothers’ waist while some UM-B discriminating bacteria were inversely associated with a reduction of anthropometric values (waist, BMI, WHR, and weight). In a previous study, comparing pregnant women with pre-pregnancy obesity to women with normal pre-pregnancy BMI, authors identified the genera *Eggerthella* and unclassified *Erysipelotrichaceae*, increased in UM-A mothers, as markers of normal weight [32]. Furthermore, gestational weight gain was associated with *Clostridium sensu stricto,* increased in UM-B mothers, while unclassified *Erysipelotrichaceae* was inversely correlated. *Christensenella* increased in UM-B mothers, which was previously reported to be associated with a higher fasting plasma glucose concentration [32]. In the same line, *Blautia* increased in UM-A mothers, which was described to be inversely associated with insulin and homeostatic model assessment of insulin resistance (HOMA-IR) values in gestational *diabetes mellitus* mothers [33]. Therefore, discriminating bacteria found in each UM could influence the host metabolism to define the anthropometric profiles found in UM-A and UM-B mothers during postpartum.

## 5. Conclusions

In conclusion, UMs determined the different restoration capacity of the gut microbiota and the anthropometric values of the mothers up to 12 months after delivery. UM-B exemplifies a resilient (unchanging) microbial profile, which would have negative connotations in this dysbiotic-prone metabotype. On the contrary, UM-A is a more elastic microbial intestinal ecology that progressively becomes normal during the year after childbirth. Our results suggest that the determination of UMs in pregnant and lactating women could be a useful tool to estimate their predisposition to the recovery of the gut microbiota and anthropometric values. We acknowledge that this is a pilot study that needs further confirmation in larger cohorts, from other geographical origins, different lifestyles (diet, physical activity, etc.), and in women (pregnant or those who have just given birth) with associated cardiometabolic risk factors. In this regard, the development of strategies to modulate the gut microbiota associated with UMs warrants further research that could influence the maternal and probably fetal health, and prevent potential future cardiometabolic risk.

## Figures and Tables

**Table 1:** Evolution of urolithin metabotypes (UMs) and enterotypes distribution in women (*n* = 32) for one year after delivery.

Volunteers (%)	Postpartum Periods			
	T1 *	T2	T3	T4
Enterotype 1	9	9	9	14
Enterotype 2	3	0	0	0
Enterotype 3	88	91	91	86
UM-0	0	0	0	0
UM-A (	50	50	53	60
UM-B	50	50	47	40
Normoweight	50.0	59.4	59.4	62.5
Overweight	37.5	31.3	31.3	25.0
Obese	12.5	9.4	9.4	12.5

* Time-points are shown in Figure 1. T1, 3 weeks (early lactation); T2, 4 months and T3, 6 months (established lactation); T4, 12 months (after lactation).

**Table 2:** Anthropometric values and α-diversity (Shannon and Chao 1 indexes) in women over one year after giving birth.

	All Women (*n* = 32)	UM-A (*n* = 16)	UM-B (*n* = 16)
BMI (kg/m^2^)			
T1 *	25.8 ± 3.5 (19.5–33.3) ^a^	25.3 ± 2.9 (22.0–30.9) ^a^	26.3 ± 4.1 (19.5–33.3) ^a^
T2	25.1 ± 3.8 (19.8–35.8) ^b^	24.5 ± 2.8 (20.8–29.2) ^ab^	25.7 ± 4.5 (19.8–35.8) ^a^
T3	24.9 ± 4.0 (19.8–36.1) ^b^	24.3 ± 2.9 (20.5–29.5) ^b^	25.5 ± 4.8 (19.8–36.1) ^a^
T4	24.3 ± 4.1 (18.7–35.8) ^c^	23.5 ± 3.3 (18.7–31.1) ^c^	25.0 ± 4.7 (18.7–35.8) ^b^
*p*-value ^ⱡ^	<0.001	0.001	0.029
Waist (cm)			
T1	92.6 ± 8.0 (76.5–109.0) ^a^	91.5 ± 6.2 (76.5–99.5) ^a^	93.6 ± 9.5 (78.7–109.0) ^a^
T2	89.4 ± 8.3 (68.0–110.0) ^b^	89.0 ± 7.2 (78.0–101.0) ^ab^	89.8 ± 9.5 (68.0–110.0) ^b^
T3	88.0 ± 9.4 (70.0–113.0) ^bc^	87.0 ± 9.0 (70.0–104.0) ^b^	89.1 ± 10.0 (76.0–113.0) ^b^
T4	85.8 ± 10.4 (65.0–115.0) ^c^	83.0 ± 8.8 (65.0–97.5)^c^	88.5 ± 11.3 (72.5–115.0) ^b^
*p*-value ^ⱡ^	<0.001	0.001	0.021
Hip (cm)			
T1	99.9 ± 8.1(86.0–116.0) ^a^	98.6 ± 6.7 (87.5–110.5) ^a^	101.2 ± 9.2 (86.0–116.0) ^a^
T2	95.7 ± 8.7 (78.0–115.0) ^b^	94.6 ± 9.1 (80.0–109.0) ^ab^	96.8 ± 8.5 (78.0–115.0) ^ab^
T3	95.7 ± 9.6 (81.0–123.0) ^b^	94.0 ± 8.7 (81.0–111.0) ^b^	97.3 ± 10.5 (83.0–123.0) ^ab^
T4	94.7 ± 11.1 (78.0–125.0) ^b^	93.1 ± 10.3 (78.0–111.5) ^b^	96.5 ± 12.2 (79.0–125.0) ^b^
*p*-value ^ⱡ^	0.010	0.060	0.107
Waist/Hip			
T1	0.93 ± 0.05 (0.75–1.02) ^ab^	0.93 ± 0.05 (0.85–1.02) ^a^	0.93 ± 0.05 (0.75–0.99) ^a^
T2	0.93 ± 0.05 (0.83–1.03) ^a^	0.94 ± 0.05 (0.83–1.03) ^a^	0.93 ± 0.04 (0.83–0.99) ^a^
T3	0.92 ± 0.04 (0.81–1.00) ^b^	0.93 ± 0.05 (0.84–1.00) ^a^	0.92 ± 0.04 (0.81–1.00) ^a^
T4	0.91 ± 0.06 (0.72–0.99) ^b^	0.89 ± 0.07 (0.72–0.98) ^b^	0.93 ± 0.04 (0.87–0.99) ^a^
*p*-value ^ⱡ^	0.025	0.026	0.203
Shannon			
T1	4.4 ± 0.9 ^a^	4.3 ± 0.1 ^a^	4.5 ± 0.1 ^a^
T2	4.4 ± 0.7 ^a^	4.3 ± 0.1 ^a^	4.4 ± 0.1 ^a^
T3	4.3 ± 0.1 ^a^	4.2 ± 0.1 ^a^	4.3 ± 0.2 ^a^
T4	4.3 ± 0.1 ^a^	4.2 ± 0.1 ^a^	4.5 ± 0.1 ^a^
*p*-value ^ⱡ^	0.607	0.749	0.659
Chao 1			
T1	2472.2 ± 132.2 ^a^	2299.6 ± 152.6 ^a^	2644.8 ± 215.9 ^a^
T2	2204.5 ± 151.9 ^a^	2129.1 ± 175.5 ^a^	2279.8 ± 248.2 ^ab^
T3	1785.2 ± 154.2 ^b^	1652.7 ± 178.1 ^b^	1917.7 ± 251.9 ^b^
T4	1848.4 ± 110.8 ^b^	1685.3 ± 127.9 ^b^	2011.5 ± 181.0 ^b^
*p*-value ^ⱡ^	0.004	0.011	0.078

* Time-points are shown in Figure 1. T1, 3 weeks (early lactation); T2, 4 months and T3, 6 months (established lactation); T4, 12 months (after lactation). Values are expressed as mean ± standard deviation (SD) and range in brackets. Values with different letters within each column are significantly different using the Bonferroni test (*p* < 0.05). ^ⱡ^ Pillai’s trace for ANCOVA analysis.
